# Core determinant of the *in vitro* enzyme activity of tenecteplase: primary structure over glycosylation modifications

**DOI:** 10.3389/fphar.2026.1827136

**Published:** 2026-06-12

**Authors:** Lyu-yin Wang, Kai-xin Xu, Jin-liang Chen, Bi-wei Bai, Hai-shu Gao, Ping Lv, Jing Li

**Affiliations:** 1 National Institutes for Food and Drug Control, Beijing, China; 2 China Pharmaceutical University, Nanjing, China; 3 Heilongjiang Institute for Drug Control, Heilongjiang, China

**Keywords:** automatic coagulation analyzer, clot lysis activity, methodological validation, reference material, standardization, tenecteplase

## Abstract

**Introduction:**

Tenecteplase (TNK), a novel genetically modified variant of tissue-type plasminogen activator (rt-PA), holds great promise as a first-line thrombolytic agent for thrombotic diseases owing to its convenient administration and favorable pharmacological properties. However, glycosylation heterogeneity derived from distinct production processes has resulted in industry-wide inconsistencies in enzyme activity determination methods, reference materials, and activity units, severely impairing the accuracy of clinical medication. Compounded by its narrow therapeutic window, minor dosage deviations of TNK can not only drastically reduce thrombolytic efficacy but also trigger severe hemorrhagic adverse reactions. This study aimed to establish an accurate and standardized *in vitro* enzymatic activity assay for TNK, and to elucidate the dominant determinant of TNK *in vitro* enzyme activity between primary structure and glycosylation modifications.

**Methods:**

An *in vitro* enzyme activity assay was established using an automatic coagulation analyzer and subjected to systematic methodological validation. The primary structures of two TNK products (TNK A and TNK B) were confirmed via LC-MS/MS, while their glycosylation profiles were characterized using HILIC coupled with mass spectrometry. Comparative activity assays were conducted under various conditions, including human serum albumin (HSA) protection, dithiothreitol (DTT) disruption, and single-chain to two-chain conversion.

**Results:**

Methodological validation demonstrated that the automated assay possesses high accuracy (relative bias −0.24%–0.27%), precision (CV < 2.0%), and robustness, with results highly consistent with the traditional bubble-rising method. Characterization confirmed that TNK A and TNK B share identical amino acid sequences but exhibit significant differences in glycan distribution. Under stable conditions (using HSA-containing buffers), no statistically significant difference in clot lysis activity was observed between the two products (P > 0.05). Experimental data revealed that previously reported activity discrepancies were likely artifacts of buffer composition (lack of HSA) and environmental sensitivity rather than direct glycan-driven functional changes.

**Discussion:**

The primary structure is the decisive factor determining the *in vitro* enzymatic activity of TNK, while glycosylation modifications appear to exert minimal impact under the specific stable *in vitro* assay conditions tested. The established automated coagulation analysis provides a reliable tool for quality control and the unification of clinical activity units for recombinant tissue-type plasminogen activator-based agents.

## 1 Introduction

Thrombosis is the common pathological basis of three major global cardiovascular diseases, including myocardial infarction, stroke, and venous thromboembolism. Characterized by high morbidity, high disability rate, and high mortality, these diseases rely on intravenous thrombolysis as the core therapeutic approach (Jolugbo and Ariëns, 2021). Thrombolytic agents dissolve fibrin clots by directly or indirectly activating plasminogen into plasmin, and have evolved into three generations to date: the first-generation agents (e.g., streptokinase, urokinase) lack fibrin specificity and are prone to inducing bleeding; the second-generation agent, recombinant tissue-type plasminogen activator (rt-PA, alteplase), has a short half-life (<5 min) and can only be administered via intravenous infusion; and the third-generation agents consist of genetically modified rt-PA variants, represented by tenecteplase (TNK) and reteplase (rPA), with extended half-lives and bolus injection feasibility (Ouriel et al., 1995; Guillermin et al., 2016; Mohammadi et al., 2019; Roaldsen et al., 2021). Among them, TNK achieves functional optimization through amino acid substitutions at three key sites, enabling bolus intravenous administration while reducing the risk of intracranial hemorrhage and minimizing clinical medication errors and time delays (Logallo et al., 2016; Li et al., 2022; Wang et al., 2024).

Tenecteplase (Metalyse®), developed by Boehringer Ingelheim International GmbH, was approved by the US FDA in 2000 for the treatment of acute ST-elevation myocardial infarction (STEMI) and is recognized as a preferred thrombolytic agent for STEMI (Melandri et al., 2009; Günlü and Demir, 2022). RhTNK-tPA (Mingfule®, CSPC Pharmaceutical Group) was launched in China in 2019. Additionally, TNK has emerged as a research hotspot in the field of intravenous thrombolysis for acute ischemic stroke (AIS). Clinical data demonstrate that TNK exhibits comparable efficacy and safety to alteplase, showing great promise as an alternative thrombolytic agent (Campbell et al., 2018; Warach et al., 2020; Berge et al., 2021; Alamowitch et al., 2023; Palaiodimou et al., 2024). As of 2024, Metalyse® has been supplied to 93 countries and regions worldwide, and the number of global TNK-related clinical trials is growing rapidly (Katsanos et al., 2021; Bivard et al., 2022; Kvistad et al., 2022; Roaldsen et al., 2023; Albers et al., 2024).

As a core quality attribute of TNK, enzyme activity is directly associated with its clinical mechanism of action, the integrity of its biological function, and batch-to-batch consistency. However, TNK products from different manufacturers exhibit significant differences in post-translational glycosylation modifications due to variations in host cells and production processes (Kliche et al., 2014; Werner et al., 2007). Consequently, various enterprises have adopted distinct glycoform-specific reference materials, established independent activity assay methods, and defined unique enzyme activity units. Despite the high catalytic activity, similar mechanisms of action, and consistent primary structure of TNK products from different manufacturers, the discrepancies mentioned above result in chaotic calibration of clinical activity units, seriously compromising the precision of clinical medication (Astrup and Müllertz, 1952; Meloni et al., 2022). Furthermore, TNK has a narrow therapeutic window: insufficient dosage results in failed vascular recanalization and irreversible damage to cardiac and cerebral tissues, whereas excessive dosage induces systemic fibrinolysis and significantly increases the risk of life-threatening hemorrhagic transformation, such as intracranial hemorrhage. Therefore, for precise clinical application, it is crucial to establish an accurate, sensitive, and efficient standardized enzyme activity assay, enabling unified calibration of enzyme activity units for TNK products with different glycoforms (Melandri et al., 2009).

In the current quality standards, the bubble-rising method is adopted for the determination of TNK enzymatic activity. This method requires special water bath equipment, is prone to large errors due to manual operation, and has low detection throughput (Appert-Flory et al., 2007; Huang, 2017; Ding et al., 2018; Li et al., 2021; Xiao et al., 2023). Although previous studies have reported that TNK products from different sources (TNK A from CSPC Pharmaceutical Group and TNK B from Boehringer Ingelheim International GmbH) exhibit differences in the single-chain to two-chain ratio, and speculated that this difference may affect the accuracy of enzymatic activity determination (Bechmann et al., 2024), the two TNK products without single-chain to two-chain conversion were not significantly different in their enzymatic activity. In the present study, we systematically investigated the effects of primary structure and glycosylation modifications on TNK enzymatic activity by conducting multidimensional characterization and analysis of the two TNK products. First, the consistency of their primary structures was verified using Orbitrap Exploris480 mass spectrometry combined with liquid chromatography–mass spectrometry (LC–MS)/MS. Second, after pretreatment, which included reduction, PNGase F digestion, and 2-AB labeling, the glycosylation profile characteristics were analyzed using hydrophilic interaction chromatography coupled with UPLC I-Class/Synapt G2-Si LC–MS. Finally, their enzymatic activities were compared using an automatic coagulation analyzer after fully converting both into the two-chain form. The results showed no significant difference in enzymatic activity after conversion, further confirming that the primary structure is the key factor determining TNK enzymatic activity.

In this study, we aim to verify the feasibility of automatic coagulation analysis as a standardized enzymatic activity assay for TNK and clarify the actual impact of glycosylation differences on its enzymatic activity We also obtained experimental results that offer a different perspective from some existing literature: under our specific assay conditions, TNK products with consistent primary sequences but different glycosylation modifications demonstrated comparable *in vitro* clot lysis activity. This finding provides a basis for the unification of TNK enzymatic activity units and offers insights that may inform future clinical evaluations.

## 2 Materials and methods

### 2.1 Materials

Tenecteplase A (TNK A) reference standard, tenecteplase A for injection and TNK A bulk solutionwere all purchased from CSPC Recomgen Pharmaceutical (Guangzhou) Co., Ltd. Tenecteplase B (TNK B) reference standard, tenecteplase B for injection, and TNK B bulk solution were obtained from Boehringer Ingelheim International GmbH (Germany). Reteplase (rPA) reference standard and reteplase for injection were purchased from Aide Pharmaceutical (Beijing) Co., Ltd. Alteplase reference standard and alteplase for injection were acquired from Boehringer Ingelheim International GmbH (Germany).

Human plasma thrombin, human plasma fibrinogen (≥90% coagulable protein), and human plasma plasminogen were purchased from EMD Millipore (United States). Peptide N-glycosidase F (PNGase F) was obtained from Agilent Technologies (United States), and another batch of PNGase F was from New England Biolabs (United States). RapiGest SF protease surfactant and 2-AB Dextran Calibration Ladder were supplied by Waters (United States). Trypsin was obtained from Roche (United States). Ultrafiltration tubes (3 kDa) were obtained from EMD Millipore (United States). The ACQUITY UPLC Peptide BEH C18 column and the ACQUITY UPLC Glycan BEH Amide column were purchased from Waters (United States).

Human serum albumin, polysorbate 20, Tween 80, L-arginine, dithiothreitol (DTT), dimethyl sulfoxide (DMSO), concentrated acetic acid, 2-aminobenzamide, sodium cyanoborohydride, isopropanol, triethylamine, sodium dodecyl sulfate (SDS), 2-mercaptoethanol, ammonium bicarbonate, iodoacetamide, formic acid, NP-40, and absolute ethanol were all purchased from Sigma Aldrich (United States). Other conventional chemical reagents were of analytical grade. Coagulation analyzer cleaning solutions A, B, and rinsing solution were purchased from Werfen (Spain).

### 2.2 Preparation of reagents

Phosphate buffer (pH 7.4) consisted of 0.0114 mol/L sodium dihydrogen phosphate dihydrate, 0.0486 mol/L disodium hydrogen phosphate dihydrate, and 0.01% (v/v) Tween 80 in purified water.

Sample dilution buffer was prepared by adding HSA to the phosphate buffer to a final concentration of 5 g/L.

Formulation buffer was composed of 0.300 mol/L L-arginine and 0.04% (v/v) Tween 20 in purified water, with the pH adjusted using 85% phosphoric acid (10 mL/L). No human serum albumin (HSA) was added to this formulation.

RapiGest SF solution and trypsin solution were reconstituted with ultrapure water to a final concentration of 1 mg/mL.The reducing agent was prepared by diluting 20% (w/v) sodium dodecyl sulfate (SDS) stock solution 10-fold with ultrapure water. Subsequently, 5% (v/v) 2-mercaptoethanol was added, and the mixture was thoroughly mixed.

The 2-AB labeling solution contained 0.367 mol/L 2-aminobenzamide and 0.955 mol/L sodium cyanoborohydride dissolved in a DMSO/acetic acid mixture (3:7, v/v).

The Adjusting solution was prepared by measuring 40% (v/v) isopropanol, 0.2% (v/v) triethylamine, and 59.8% (v/v) ultrapure water, followed by thorough mixing.

The DTT solution was prepared by dissolving DTT in ultrapure water and adjusting the volume to achieve a final concentration of 0.0207 mol/L.

### 2.3 Protein determination, establishment and validation of the automated clot lysis assay

For TNK samples, absorbance differences at 280 nm and 320 nm were measured using an ultraviolet spectrophotometer, and protein concentration was calculated with an extinction coefficient of 1.9 mL/(mgcm). The samples were serially diluted with sample dilution buffer to final working concentrations of 800, 1,000, and 1,200 ng/mL. Additionally, the TNK A reference standard was reconstituted with water to 0.527 mg/mL, and followed by dilution to generate linear standard solutions ranging from 600 to 1,400 ng/mL.

The enzyme activity was determined using an ACL TOP 700 automatic coagulation analyzer (Instrumentation Laboratory, Bedford, MA, United States). The mixed solution was used as the starting reagent, and human plasma thrombin was used as the intermediate reagent. The sample/reference standard, starting reagent, and intermediate reagent volumes were set to 20, 200, and 20 μL, respectively. The clot lysis time was measured at 405 nm at 37 °C. Linear regression was performed with the logarithm of lysis time as the ordinate (Y-axis) and the logarithm of the reference standard concentration as the abscissa (X-axis) to establish the standard curve equation. The mean concentration of the test sample was calculated by substituting the measured lysis time of the sample into the validated standard curve equation. The enzyme activity of the sample was then calculated using the following formula: Enzyme activity (IU/mL) = mean protein concentration (mg/mL) × specific activity of the reference standard (IU/mg).

To ensure method reliability, a systematic methodological validation was performed encompassing specificity, accuracy, precision, repeatability, and robustness. Specificity was confirmed by testing the formulation buffer and a solution with a molar concentration equivalent to HSA to rule out any interference from non-target components. Accuracy and intermediate precision were thoroughly evaluated across five potency levels (60%, 80%, 100%, 120%, and 140% of the target concentration, corresponding to 600–1,400 ng/mL). These levels were analyzed by two independent analysts over two consecutive days, with two technical replicates per concentration per analyst per day (n = 8 per level), utilizing relative bias (RB) for accuracy, and the coefficient of variation (CV) alongside the 95% confidence interval upper limit (CICV) for precision. Repeatability was established by analyzing six parallel preparations of TNK for injection twice each. Finally, robustness was verified by evaluating the stability of the sample solution stored in the analyzer for 0, 3, and 5 h, and by investigating activity changes under deliberate variations of the fibrinogen-to-plasminogen ratio (45:1, 50:1, and 55:1). The validated automated method was used to compare the clot lysis activities of three batches each of TNK A and TNK B (both bulk and formulations) against their respective reference standards. To investigate buffer matrix effects, a parallel assay was conducted using an HSA-free dilution buffer.

### 2.4 Comparative clot lysis time determination of multiple rt-PA-based thrombolytic agents

Alteplase, TNK A and reteplase were diluted to a uniform molar concentration of 17.03 nmol/L (calculated from 1,000 ng/mL TNK A, theoretical molecular weight 58,718 Da) with sample dilution buffer, using theoretical molecular weights of 59,042 Da for alteplase and 39,571 kDa for reteplase for concentration calculation. The clot lysis time of each sample was determined in strict accordance with the method described in Section 2.3 using an ACL TOP 700 automatic coagulation analyzer, with six independent parallel replicates per sample.

### 2.5 Determination of TNK activity using the bubble-rising method

One vial of TNK A reference standard was reconstituted according to the manufacturer’s instructions and serially diluted with sample dilution buffer to prepare standard solutions with final concentrations of 2,750, 1,375, 687.5, 343.75, 171.88, and 85.9375 IU/mL. After reconstitution, TNK A for injection and TNK A bulk solution were measured to determine the protein concentration according to Section 2.3, before diluting with sample dilution buffer to 1 μg/mL as test solutions.

Next, solution B (1 mL of human plasma fibrinogen solution mixed with 20 μL of human plasma plasminogen solution) was added to a test tube, before adding 0.2 mL of solution A (150 μL of test/standard solution mixed with an equal volume of human plasma thrombin solution), and incubating in a 37 °C water bath for timing. The reaction system coagulated within 30 s, and the time when the last small bubble in the clot rose to the solution surface was recorded as the endpoint. To calculate the TNK activity (IU/mL), linear regression was performed, with the logarithm of TNK reference standard concentration (IU/mL) as the abscissa and the logarithm of endpoint time (s) as the ordinate to calculate the TNK activity (IU/mL).

### 2.6 Determination of the amino acid sequences of TNK A and TNK B

TNK A and TNK B reference standards were diluted to 1 mg/mL with 50 mmol/L ammonium bicarbonate and concentrated by ultrafiltration using a 3-kDa ultrafiltration tube. A solution containing 100 μg of protein was mixed with RapiGest SF for dissolution, then subjected to DTT reduction, iodoacetamide alkylation in the dark, PNGase F deglycosylation, and trypsin digestion. The reaction was terminated by adding 10% formic acid for sample preparation.

Separation was performed on an ACQUITY UPLC Peptide BEH C18 column with a gradient elution of 0.1% formic acid/water (phase A) and 0.1% formic acid/acetonitrile (phase B). Mass spectrometry was conducted in the ESI source in the full scan-ddMS2 positive ion mode with HCD fragmentation. The theoretical sequence of TNK was input into BioPharma Finder software, with iodoacetamide alkylation as a fixed modification and deamidation as a variable modification, to match peptides and fragments for sequence identification. All experiments were performed in three independent biological replicates (n = 3), with two technical replicates per sample.

### 2.7 Glycan profiling of TNK A and TNK B

TNK A and TNK B samples were first subjected to reduction and denaturation at 90 °C for 10 min using a reducing agent, followed by the addition of 7.5% Triton X-100. The N-glycans were enzymatically released by incubation with PNGase F at 37 °C for 2 h. The liberated glycans were then fluorescently labeled with 2-AB at 65 °C for 2 h in the dark. Subsequently, the labeled glycans were purified using Supelclean ENVI-Carb solid-phase extraction (SPE) cartridges, vacuum dried, and reconstituted in a water-acetonitrile mixture for analysis.

Chromatographic separation was achieved on an ACQUITY UPLC Glycan BEH Amide column (150 mm × 2.1 mm, 1.7 μm) maintained at 60 °C. Mobile phase A consisted of 50 mmol/L ammonium formate (50% acetonitrile, v/v, pH 4.5 ± 0.05), and mobile phase B was 100% acetonitrile. The elution gradient was programmed as follows: 0–0.3 min, 56%–50% B; 0.3–40 min, 50%–15% B; 40–41 min, 15%–56% B; and 41–46 min, hold at 56% B. The flow rate was set at 0.6 mL/min. Detection was performed using a fluorescence detector with excitation and emission wavelengths set at 330 nm and 420 nm, respectively. Mass spectrometry analysis was conducted in ESI positive ion mode, and glycan quantification was performed using UNIFI software.

### 2.8 Determination of clot lysis activity of TNK A and TNK B after single-chain to two-chain conversion

TNK A and TNK B samples were first subjected to buffer exchange via ultrafiltration, and their protein concentrations were uniformly adjusted to 0.4 mg/mL. The conversion reaction was initiated by adding 10 μL of human plasma plasminogen to 500 μL of the sample, followed by incubation at 37 °C. At 30, 60, and 120 min, 20 μL aliquots were collected and immediately mixed with 780 μL of DTT solution (20.7 mmol/L) to terminate the conversion, and subsequently diluted to 1,000 ng/mL for analysis. To systematically evaluate the relationship between the two-chain ratio and enzymatic activity, the native TNK A sample (containing approximately 10% two-chain form) was utilized as a baseline, and a 100% two-chain variant was prepared via complete plasminogen-induced cleavage (37 °C for 1 h). Gradient samples with theoretical two-chain ratios of 30%, 50%, 70%, and 90% were generated by mixing the baseline sample and the 100% two-chain variant at specific volume ratios. The actual two-chain proportions of each sample were verified using reduced high-performance size-exclusion chromatography (HP-SEC).

The clot lysis activity of all samples was determined using the ACL TOP automated coagulation analyzer, according to the protocol described in Section 2.3. For comparative analysis, data were normalized by setting the activity of the native baseline sample to 100%. The experiment was performed with three independent biological replicates.

### 2.9 Effects of HSA and DTT on the thermal denaturation temperature (Tm) of TNK A and TNK B

The Tm of different samples was determined using a MicroCal PEAQ-DSC automated differential scanning calorimeter (Malvern Panalytical, United Kingdom). To investigate the effects of different additives, three treatment groups were established:

Control group: TNK A and TNK B reference standards were purified by ultrafiltration with formulation buffer (12,000 rpm for 15 min, repeated twice), diluted to 5 mg/mL, and further diluted to 0.025 mg/mL with formulation buffer.

DTT treatment group: The above standards were purified by ultrafiltration with formulation buffer, diluted to 5 mg/mL, and then diluted to 0.05 mg/mL with phosphate buffer, before adding 20.7 mmol/L DTT solution to adjust the final TNK concentration to 0.025 mg/mL.

HSA + DTT treatment group: The above standards were purified by ultrafiltration with formulation buffer, diluted to 5 mg/mL, and then diluted to 0.05 mg/mL with phosphate buffer containing 5 g/L HSA, before adding 20.7 mmol/L DTT solution to adjust the final concentration to 0.025 mg/mL.

The corresponding dilution buffer of each group was used as the blank control. The sample plate was loaded into the instrument sample cell, and the scanning program was set as follows: initial temperature, 25 °C; heating rate, 1.5 °C/min; and final temperature, 130 °C. The Tm value of the sample was determined by fitting the peak vertex temperature of the thermal denaturation curve. For quantitative comparison, thermal stability was expressed as a normalized Tm, calculated as (Tm_sample_/Tm_control_) × 100%. All determinations were performed in biological triplicates with two technical replicates each.

## 3 Results

### 3.1 Methodological validation of automatic coagulation analysis for the determination of TNK activity

**FIGURE 1:**
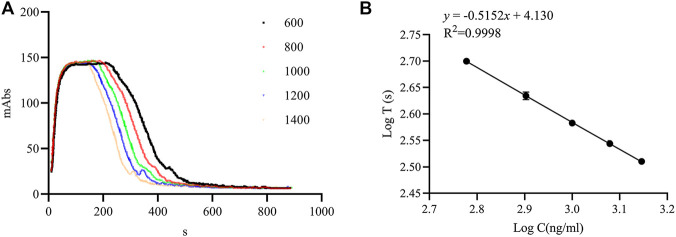
Establishment of the tenecteplase (TNK) assay system using an automatic coagulation analyzer. **(A)** Representative clot lysis curves for a concentration series of TNK A within the validated range (600, 800, 1,000, 1,200, and 1,400 ng/mL). The decrease in absorbance (mAbs) indicates the dissolution of the fibrin clot over time. **(B)** Standard curve of TNK A established using automatic coagulation analysis. Linear regression for the standard curve was performed with the logarithm of lysis time as the ordinate and the logarithm of concentration as the abscissa.

Systematic methodological validation was performed, covering specificity, accuracy, precision, linearity and range, repeatability, and robustness. As shown in Figure 1A, the reaction mixture of TNK A samples exhibited a typical clot lysis curve at 37 °C, whereas no clot lysis was detected in HSA or formulation buffer (data not shown), indicating the high specificity of the method. The standard curve for TNK A was established by plotting the logarithm of lysis time against the logarithm of concentration (Figure 1B). This regression analysis confirmed a strict dose-response relationship, indicating that the automated analyzer can sensitively and linearly capture changes in TNK concentration.

**FIGURE 2:**
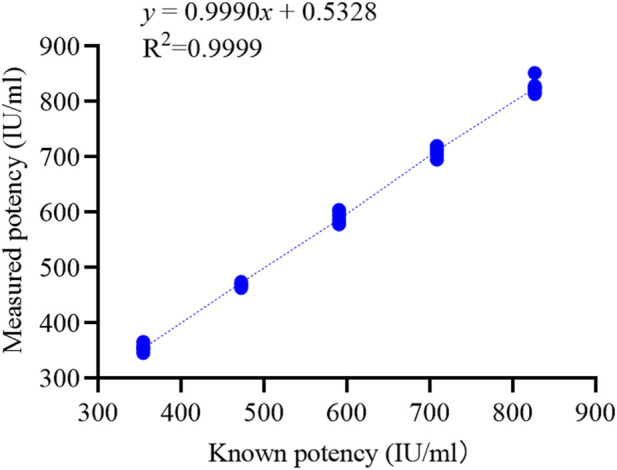
Linearity analysis of tenecteplase enzyme activity determination using automatic coagulation analysis. *The linear regression equation was Y = 0.9990X + 0.5328, with *R* ^2^ = 0.9999 for the measured and theoretical potencies.

Two analysts measured TNK samples, with potency levels ranging from 60% to 140% over 2 consecutive days, with eight replicates per concentration (Table 1). The results showed that the RB ranged from −0.24% to 0.27%, the CV for each potency level was <2.0%, and the 95% CICV was <4.0%. As shown in Figure 2, the method exhibited good accuracy, intermediate precision, and linearity across the validated concentration range. For repeatability validation, six independent parallel sample solutions of TNK for injection were prepared synchronously with the same standard curve (biological replicates, n = 6), and each solution was measured twice consecutively (technical replicates, n = 2 per sample), resulting in a total of 12 independent determinations. The average potency from the 12 determinations was 1,003 320.67 IU/vial, with a coefficient of variation (CV) of 1.83%, confirming the satisfactory repeatability of the established method. In robustness validation, the CV of the determination results was <3% when the sample solution was placed in the coagulation analyzer for 0, 3, and 5 h, and when the ratios of fibrinogen to plasminogen were 45:1, 50:1, and 55:1, indicating the method’s robustness.

**TABLE 1:** Accuracy and intermediate precision at five potency levels estimated using the automatic coagulation analyzer.

Potency level	Run	Bioactivity (IU/mL)			Accuracy (%)			Intermediate precision (%)	
		Mean	Lower confidence	Upper confidence	Relative bias	Lower confidence	Upper confidence	CV	CICV
60%	8	311,839.02	307,687.27	315,990.77	0.27%	−1.07%	1.60%	1.99%	3.58%
80%	8	310,555.81	306,584.22	314,527.41	−0.14%	−1.42%	1.13%	1.91%	3.44%
100%	8	310,265.45	306,184.99	314,345.90	−0.24%	−1.55%	1.08%	1.97%	3.53%
120%	8	311,774.90	307,987.86	315,561.95	0.25%	−0.97%	1.47%	1.82%	3.26%
140%	8	310,686.33	306,959.54	314,413.13	−0.10%	−1.30%	1.10%	1.80%	3.22%

### 3.2 Clot lysis time of rt-PA-based thrombolytic agents

**FIGURE 3:**
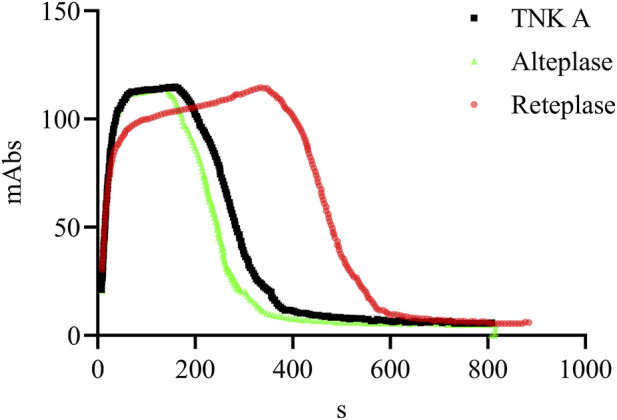
The curves show the dynamic changes of absorbance (mAbs) at 405 nm during fibrin clot formation and lysis at 37 °C. Black square: tenecteplase A (TNK A); green triangle: alteplase; red dot: reteplase. All samples were adjusted to an equimolar concentration (∼17.04 nmol/L) for the assay.

Under equimolar concentration, the average clot lysis time of alteplase, TNK A and reteplase was 345.2 ± 4.8 s, 400.4 ± 5.1 s and 531.6 ± 7.3 s (Figure 3). The established automated assay distinguished differences in fibrinolytic activity among the rt-PA variants.

### 3.3 Comparison between automatic coagulation analysis and the classic bubble-rising method

**FIGURE 4:**
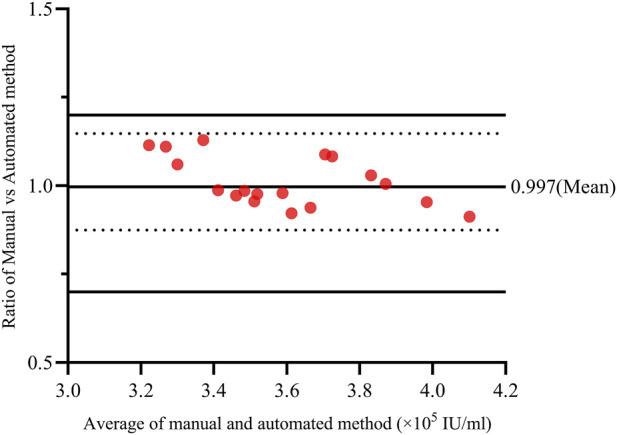
Bland-Altman consistency analysis of tenecteplase activity results determined using automatic coagulation analysis and the manual bubble-rising method. The dotted lines represent the actual 95% limits of agreement (0.875–1.148). The bold solid lines represent the predefined acceptable equivalence margins, precisely located at 0.7 and 1.2. All data points fell within both the statistical limits of agreement and the predefined equivalence margins, confirming the reliability of the automated method.

The enzyme activities of six batches of TNK A formulations and bulk solutions were determined using both methods. The Shapiro–Wilk test indicated that both sets of data followed a normal distribution, and the paired *t*-test results showed that all *P*-values were >0.05 (Table 2), demonstrating no statistical difference between the two methods. The mean ratio of the results obtained by automatic coagulation analysis to those by the bubble-rising method was 1.01, and all data points fell within the 95% confidence interval (0.875–1.148) (Figure 4), confirming a high level of agreement the two methods. Additionally, the CV of the automatic coagulation analysis method was significantly lower than that of the bubble-rising method, indicating superior precision.

**TABLE 2:** Comparison of tenecteplase activity as determined by automatic coagulation analysis and the bubble-rising method.

Batch number	Automatic coagulation analysis		Bubble-rising method		*P-*value
	Average (IU/mL)	CV (%)	Average (IU/mL)	CV (%)	
20230715T	339,948.95	0.35	333,088.00	7.43	0.67
20230729T	346,067.90	0.55	347,295.24	9.78	0.95
20230724T	342,866.15	0.89	343,451.30	5.94	0.96
A24072001P	387,754.52	0.37	382,923.64	6.95	0.77
B24080801P	389,035.87	0.53	388,030.48	9.49	0.96
C24080901P	355,721.44	0.42	352,524.57	9.08	0.88

### 3.4 Amino acid sequence analysis of TNK samples

To confirm their primary structures, complete mass spectrometry analysis of trypsin-digested TNK A and TNK B reference standards was performed using LC–MS/MS. The peptide matching and amino acid sequences of TNK from the two manufacturers were identical, with a sequence coverage of 100% for both.

### 3.5 Glycan profiling of TNK samples

**FIGURE 5:**
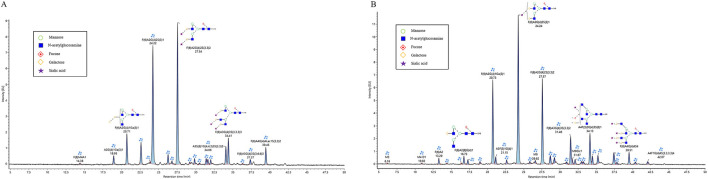
Hydrophilic interaction chromatography (HILIC) profiles of tenecteplase reference standards with distinct glycosylation modifications. **(A)** Tenecteplase A (Mingfule®) reference standard. **(B)** Tenecteplase B (Metalyse®) reference standard. *The main glycan structures and their relative abundances are labeled for characteristic peaks.

Glycosylation modifications of TNK A and TNK B were characterized using UPLC–FLD–MS (Figure 5). The main glycan structures of both included core fucosylated biantennary structures and complex N-glycans with 1–2 terminal sialic acids. The common glycans with an abundance of >10% were F(6)A2G(4)1Ga(3)1, F(6)A2G(4)2S(3)1, and F(6)A2G(4)2S(3,3)2. Among them, the peak area ratio of F(6)A2G(4)1Ga(3)1 in TNK A was approximately 8% lower than that in TNK B, whereas the peak area ratio of F(6)A2G(4)2S(3,3)2 in TNK A was approximately 16% higher than that in TNK B. Significant differences were observed in glycans with a retention time of 30–45 min: the main glycans in TNK A were A3S(6)1G(4,4,3)3S(3,3)2, F(6)A3G(4)3S(3,3,3)3, and F(6)A4G(4)4Lac1S(3,3)2, accounting for 14% of the total glycan content; the main glycans in TNK B were F(6)A3G(4)3S(3,3)2, A4F(3)2G(4)3S(6)1, and F(6)A3G(4)3S(3,3,6)3, accounting for 15% of the total glycan content (Tables 3, 4).

**TABLE 3:** Overview of the glycosylation characterization of tenecteplase A (Mingfule^®^).

Component name	Observed RT (min)	Expected glycan units	Glycan units	Expected mass (Da)	Observed mass (Da)	Area (%)
A2G (4)1Ga (3)1	18.93	7.1013	7.0962	1760.66	1760.66	1.94
F (6)A2G (4)1Ga (3)1	20.71	7.505	7.478	1906.72	1906.72	6.65
A2G (4)2S (3)1	22.62	7.9809	7.9013	2051.76	2051.76	5
F (6)A2G (4)2S (3)1	24.22	8.3282	8.2696	2,197.81	2,197.81	29.29
A2G (4)2S (3.3)2	26.25	8.8245	8.7594	2,342.85	2,342.85	2.26
F (6)A2G (4)2S (3.3)2	27.54	9.1059	9.0837	2,488.91	2,488.91	32.09
A2G (4)2S (6.6)2	29.85	9.6669	9.69	ND	ND	1.25
A3G (4)3GlcNAc3 iso2	31.42	10.1111	10.1215	ND	ND	1.62
F (6)A2BG (4)2S (6.6)2	31.64	10.1752	10.1818	ND	ND	1.26
A3S (6)1G (4,4,3)3S (3.3)2	34.06	10.844	10.8796	ND	ND	4.05
F (6)A3G (4)3S (3,3,3)3	34.41	11.14	10.9836	3,145.14	3,145.14	5.91
F (6)A3G (4)3S (3,6,6)3	37.37	11.886	11.8961	ND	ND	1.39
F (6)A4G (4)4Lac1S (3.3)2	39.45	12.58	12.5853	ND	ND	3.95

ND: not detected.

**TABLE 4:** Overview of the glycosylation characterization of tenecteplase B (Metalyse^®^).

Component name	Observed RT (min)	Expected glycan units	Glycan units	Expected mass (Da)	Observed mass (Da)	Area (%)
F (6)A2	13.29	5.8741	5.9329	1,582.61	1,582.5	1.03
F (6)A2 [6]G (4)1	16.73	6.7	6.6206	1744.67	1744.55	1.36
F (6)A2G (4)1Ga (3)1	20.73	7.505	7.4342	1906.72	1906.59	14.67
A3F (3)1G (4)1	21.15	7.5163	7.5263	ND	ND	1.38
F (6)A2G (4)2S (3)1	24.24	8.3282	8.2429	2,197.81	2,197.66	29.38
F (6)A2G (4)2S (3.3)2	27.57	9.1059	9.1012	2,488.91	2,488.74	15.79
A4G (4)2GLcNAc2	28.67	9.1111	9.4001	ND	ND	2.03
F (6)A3G (4)3S (3)1	29.21	9.6386	9.5508	2,562.95	2,562.79	1.29
F (6)A3G (4)3S (3.3)2	31.46	10.3162	10.1792	2,854.04	2,853.83	5.77
A4F (3)2G (4)3S (6)1	34.13	10.872	10.9369	1,457.05	ND	5.88
F (6)A3G (4)3S (3,3,3)3	34.46	11.14	11.0318	3,145.14	3,144.92	1.61
A4G (4)4Lac1	35.22	11.1955	11.251	ND	ND	1.7
F (6)A3G (4)3S (3,3,6)3	37.42	11.886	11.8935	ND	ND	2.78
F (6)A4G (4)4S4	39.51	12.77	12.5508	3,801.36	3,801.12	2.21

Our glycan profiling results showed overall consistent trends in major glycan features with the findings of Bechmann et al. (2024). Minor discrepancies in exact relative abundance values were attributed to the distinct analytical strategies: our study used a released-glycan HILIC-FLD-MS workflow for global glycan profiling, while the previous report employed a site-specific glycopeptide LC-MS approach.

### 3.6 Determination of the enzyme activity of TNK products with different glycosylation modifications via automatic coagulation analysis

**FIGURE 6:**
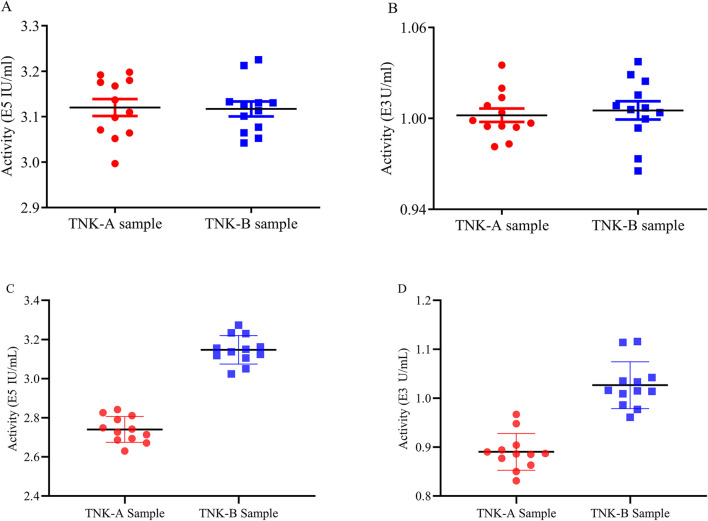
Clot lysis activity of 12 batches of tenecteplase products with two distinct glycosylation modifications quantified by automatic coagulation analysis **(A)** Determination with tenecteplase A (Mingfule®) as the reference standard in an HSA-containing buffer. **(B)** Determination with tenecteplase B (Metalyse®) as the reference standard in an HSA-containing buffer. No statistically significant difference was observed between the two TNK products under HSA protection (*P > 0.05*). **(C)** Determination with tenecteplase A as the reference standard in an HSA-free dilution buffer. **(D)** Determination with tenecteplase B as the reference standard in an HSA-free dilution buffer. *In the absence of HSA, TNK A exhibited significantly lower activity compared to TNK B (*P < 0.05*).

The clot lysis activities of three batches each of TNK A bulk solution and TNK A formulation, and three batches each of TNK B bulk solution and TNK B formulation were determined using TNK A and TNK B as reference standards, respectively, according to the method described in Section 2.3 (Figures 6A,B). When TNK A was used as the reference standard, the paired *t*-test *P*-value for the activities of the two TNK products was 0.83; when TNK B was used as the reference standard, the P-value was 0.68 (both >0.05). Clot lysis activities were also evaluated using an HSA-free dilution buffer (Figures 6C,D). Under these unprotected conditions, a statistically significant difference emerged between the two products. Whether using TNK A or TNK B as the reference standard, TNK A exhibited significantly lower apparent *in vitro* clot lysis activity compared to TNK B (P < 0.05), representing an activity reduction of approximately 13%.

### 3.7 Determination of the clot lysis activity of TNK A and TNK B in the two-chain form

**FIGURE 7:**
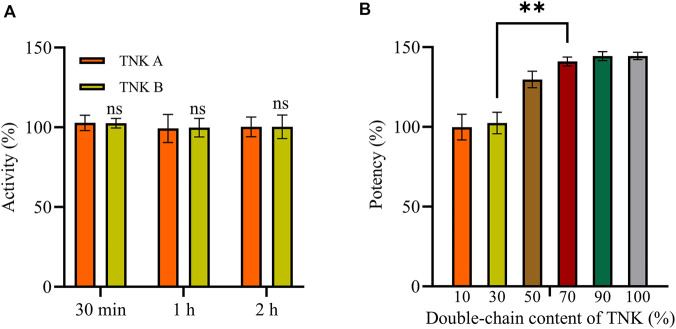
Clot lysis activity analysis of tenecteplase after plasminogen-induced single-chain to two-chain conversion. **(A)** Clot lysis activity of tenecteplase A (Mingfule®) and tenecteplase B (Metalyse®) after plasminogen treatment for different time periods (ns, no significant difference). **(B)** Clot lysis activity of tenecteplase at different two-chain ratios. A significant difference in clot lysis activity was observed between the 30% and 70% two-chain groups (P = 0.0037).

The clot lysis activity of TNK during single-chain to two-chain conversion was analyzed, with the results shown in Figure 7. The enzymatic activity was monitored during the plasminogen-induced conversion of TNK from the single-chain to the two-chain form (Figure 7A). To ensure clarity, the activity of the uncleaved baseline sample (0 min) was set as 100%. No significant difference in clot lysis activity was observed between TNK A and TNK B at any stage of the 120-min conversion process (*P > 0.05*).

Using TNK A as a model, we prepared gradient samples with two-chain ratios of 30%, 50%, 70%, 90%, and 100%. Normalization analysis, with the activity of the 10% two-chain sample set to 100%, showed that the activity of the 30% two-chain group was 102.45% (no significant difference from the 10% group, *P > 0.05*), whereas the activities of the 50%, 70%, 90%, and 100% two-chain groups were 129.73%, 141.02%, 144.38%, and 144.48%, respectively (all significantly higher than the 10% group, *P < 0.05*) (Figure 7B).

### 3.8 TNK Tm determination results

**FIGURE 8:**
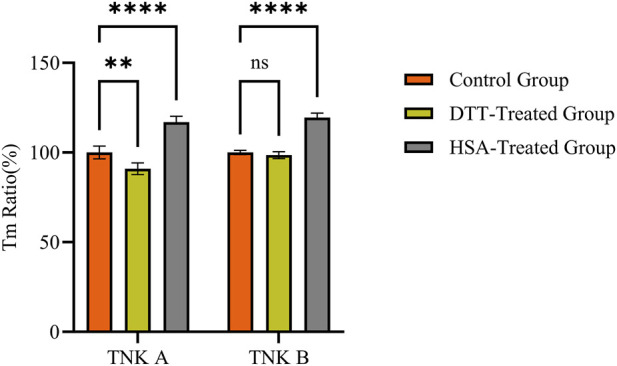
Regulatory effects of human serum albumin (HSA) and dithiothreitol (DTT) on the thermal denaturation temperature (Tm) of tenecteplase A (Mingfule®) and tenecteplase B (Metalyse®). *ns: No significant difference; **P < 0.01; ****P < 0.0001 vs. the control group. The normalized Tm value of the control group was set to 100%.

The effects of HSA and DTT on the thermal stability of TNK A and TNK B were investigated. Three treatment groups were established, including the control group (without HSA and additional DTT), DTT treatment group, and HSA + DTT treatment group. For normalization analysis, the Tm of the untreated control group was set to 100%, and the normalized Tm value was defined as the ratio of the Tm of the treated sample to the Tm of the control group (Figure 8).

The normalized Tm value of TNK A decreased to 90.8% (significant difference from the control group, *P < 0.01*), while that of TNK B decreased to 98.5% (no significant difference, ns, *P > 0.05*). After the addition of HSA, the normalized Tm values of TNK A and TNK B increased to 116.9% and 119.4%, respectively (both extremely significant differences from the control group, ****P < 0.0001).

## 4 Discussion

TNK enzymatic activity is directly associated with *in vivo* thrombolytic efficacy. Given the narrow therapeutic window of TNK, dosage deviations often lead to insufficient efficacy or life-threatening hemorrhage. Therefore, establishing an accurate and standardized enzyme activity assay is crucial to ensuring TNK quality control and precision in clinical medication (Dean et al., 2017; Liu et al., 2023). At present, TNK quality control faces two major challenges: First, the conventional bubble-rising method is hindered by significant manual operation errors, low throughput, and inadequate accuracy (Appert-Flory et al., 2007; Huang, 2017; Ding et al., 2018; Li et al., 2021; Xiao et al., 2023). Second, TNK products from different manufacturers exhibit distinct post-translational glycosylation profiles due to variations in host cells and production processes, leading to inconsistencies in activity assay methods, reference materials, and enzyme activity units across the industry (Astrup and Müllertz, 1952; Kliche et al., 2014; Meloni et al., 2022). For example, TNK A (Mingfule®), TNK B (Metalyse®), and EIAxim (India) are all expressed in CHO cells, share an identical primary sequence, and have the same clinical dosage and administration routes. Clinical data have established their clinical equivalence (Kliche et al., 2014; Logallo et al., 2017; Wang et al., 2023); however, due to differences in glycosylation modifications and assay systems, their specific activity calibrations vary drastically (6.25 × 10^5^ IU/mg vs. 200 U/mg vs. 0.02 U/mg), thus severely hindering the consistency of clinical medication.

To address these issues, an *in vitro* enzyme activity assay for TNK using an automatic coagulation analyzer was established and validated. During the optimization of sample dilution buffers, the recovery rate of TNK in simple phosphate buffer or formulation buffer was less than 80%. As a macromolecular stabilizer, HSA maintains enzyme stability during high-fold dilution by reducing non-specific adsorption to the reaction system and stabilizing protein conformation against environmental disturbances (Barbosa et al., 2010; Faroongsarng and Kongprasertkit, 2014; Frahm et al., 2012). The results of the methodological validation demonstrated that the assay possesses highspecificity, accuracy, precision, and robustness, with results that are highly consistent with those obtained using the traditional bubble-rising method but with superior precision.

In addition, the method can sensitively distinguish differences in enzymatic activity among alteplase, reteplase, and TNK, making it suitable for quality control of rt-PA-based thrombolytic agents and providing a reliable tool for the standardization of TNK activity determination.

Comparative analysis of *in vitro* clot lysis times among three rt-PA drugs revealed that alteplase < TNK A < reteplase. This difference may be attributed to molecular structural characteristics, in that reteplase lacks the Finger, EGF-like, and Kringle 1 domains, whereas TNK contains three key amino acid mutations. These structural changes result in differences in fibrin-binding capacity, thereby affecting the thrombolytic rate (Keyt et al., 1994). This study confirmed that the automatic coagulation analyzer can effectively capture this structure–activity relationship, further verifying the scientificity and applicability of the method.

LC–MS/MS and glycan profiling confirmed that TNK A and TNK B share identical primary structures but exhibit significant differences in glycosylation modifications (e.g., abundance of major glycan chains and types of high-retention-time glycans). However, determinations using the automatic coagulation analyzer showed no statistically significant differences in clot lysis activity between the two products, irrespective of whether single- or two-chain conversion was performed. This contradicts the conclusion proposed by Jan Bechmann et al. (2024) that glycosylation modifications and single-chain or two-chain ratios cause TNK A to have lower activity than TNK B (Bechmann et al., 2024). To resolve this controversy, targeted validation was conducted: when both TNK products were subjected to ultrafiltration buffer exchange with TNK B formulation buffer and assayed using HSA-free dilution buffer, TNK A exhibited 13% lower activity than TNK B, consistent with the literature results. Conversely, when HSA-containing dilution buffer was used and the products were converted to the two-chain form, their activities were highly consistent (Parcq et al., 2012).

Furthermore, DTT disruption experiments and thermal stability determinations revealed the underlying mechanism of the observed differences. TNK A exhibits higher sensitivity to the reducing agent DTT than TNK B in the absence of HSA protection, as evidenced by a significant decrease in its thermal denaturation temperature (Tm) to 90.8% of the control value, while TNK B remains relatively stable (Tm at 98.5%). Importantly, the addition of HSA effectively normalized the environmental sensitivity of both products, significantly enhancing their thermal stability (increasing normalized Tm to 116.9% and 119.4%, respectively). These findings suggest that the previously reported activity discrepancies are likely artifacts of environmental sensitivity—specifically, the differential susceptibility of certain glycoforms to conformational disturbances when protective stabilizers like HSA are absent (Duskey et al., 2020; Okochi et al., 2007).

Despite the successful establishment of a standardized assay, several limitations of this study should be noted. First, while the HSA-stabilized system accurately quantifies the core catalytic activity, its stabilizing effect may mask subtle surface interaction differences between glycosylation variants. Additional, structural disruption observed during PNGase F treatment in our pre-experiments precluded a direct, causal verification of independent glycan effects on enzymatic activity. Finally, this simplified *in vitro* model cannot fully replicate the physiological complexity of human circulation. Notably, glycosylation modifications may play critical roles in TNK’s *in vivo* biological behaviors, including plasma clearance rate, structural stability under physiological stress, immunogenicity, and interaction with cell surface receptors, which collectively determine its pharmacokinetic characteristics, *in vivo* efficacy, and clinical safety (Høiberg-Nielsen et al., 2009). Therefore, our findings—which are currently based on two globally prominent commercial TNK products—require further *in vivo* validation and generalization across a broader panel of glycoform variants.

In summary, the automatic coagulation analysis method established in this study demonstrated strong specificity, high accuracy and precision, and results consistent with those of the traditional bubble-rising method, while offering superior performance. Additionally, this study shows that the primary structure is the primary determinant for TNK enzymatic activity and suggests that glycosylation modifications exert minimal effects on *in vitro* enzymatic activity under appropriately stable conditions. These findings provide a new experimental perspective on the ongoing industry discussions regarding the impact of glycosylation on TNK activity. In the future, TNK substances with consistent primary structures may serve as unified reference standards to achieve uniform calibration of enzyme activity units for TNK products with different glycoforms. This strategy provides technical support for new drug development, process optimization, and TNK quality control, and lays the foundation for establishing a standardized TNK enzyme activity assay system, which may ultimately support more consistent clinical assessments.

## Data Availability

The datasets presented in this study can be found in online repositories. All raw experimental data have been deposited in the Zenodo repository: https://doi.org/10.5281/zenodo.20602514.
